# Effect of Consumers’ Acceptance of Indigenous Leafy Vegetables and Their Contribution to Household Food Security

**DOI:** 10.3390/su15064755

**Published:** 2023-03-07

**Authors:** Mjabuliseni Simon Cloapas Ngidi, Sinethemba Sibusisiwe Zulu, Temitope Oluwaseun Ojo, Simphiwe Innocentia Hlatshwayo

**Affiliations:** 1 Department of Agricultural Extension and Rural Resource Management, School of Agricultural, Earth and Environmental Sciences, College of Agriculture, Engineering and Science, University of KwaZulu-Natal, Private Bag X01, Scottsville, Pietermaritzburg 3201, South Africa; 2 Centre for Transformative Agricultural and Food Systems, School of Agricultural, Earth and Environmental Sciences, College of Agriculture, Engineering and Science, University of KwaZulu-Natal, Private Bag X01, Scottsville, Pietermaritzburg 3201, South Africa; 3 African Centre for Food Security, School of Agricultural, Earth and Environmental Sciences, College of Agriculture, Engineering and Science, University of KwaZulu-Natal, Scottsville, Pietermaritzburg 3201, South Africa; 4 Department of Agricultural Economics, Obafemi Awolowo University, Ile-Ife 220101, Nigeria; 5 Disaster Management Training and Education Centre for Africa, University of the Free State, Bloemfontein 9301, South Africa

**Keywords:** indigenous leafy vegetables, households, food security, endogenous switching regression model

## Abstract

In the past decades, indigenous leafy vegetables (ILVs) have played a significant role in household food security, especially in poor rural households. However, ILVs have been replaced by exotic cash crops in the contemporary world. This study was conducted to assess the consumption of indigenous leafy vegetables and their contribution to household food security of households in Limpopo and Mpumalanga provinces. The study used secondary data collected by the South African Vulnerability Assessment Committee in 2016. A total of 1520 respondents were selected using a multistage sampling method. The results from descriptive statistics revealed that most consumers did not produce ILVs but consumed them. Meanwhile, a small number of people produced ILVs yet did not consume them. The results from the Household Food Insecurity Access Scale (HFIAS) showed that a large proportion of the population experienced moderate food security while some of the individuals within the population experienced severe food insecurity. An endogenous switching regression model (ESRM) was employed to analyze the impact of the consumption of ILVs on household food security. The results revealed that only a few variables of the consumption of ILVs were significant and positive (household size, wealth index, and ‘if the disabled person receives grants’). As a result, the consumption of ILVs had a minimal impact on the household food security of the Limpopo and Mpumalanga provinces. The findings further revealed that age, gender, and education variables negatively influenced the consumption of ILVs. Thus, the recommended programs must be established to educate people about the importance of consuming ILVs. Agricultural extension services must equally promote the consumption of exotic cash crops and ILVs. Lastly, policies can contribute by increasing the diversity of ILVs left at retail outlets through diverse production.

## 1 Introduction

South Africa is one of the countries regarded as food secure at a national level, producing enough food to adequately feed its population of 53 million [1]. However, a relatively large number of people at a household level are food insecure and live below the poverty line [2]. This is evident in that many people at a household level suffer from malnutrition and hidden hunger. This has resulted in micronutrient deficiency diseases caused by hidden hunger [3]. According to Uauy et al. [4], 204 million people in developing countries suffer from micronutrient deficiency diseases. Another research conducted by the South African National Health and Nutrition Examination Survey (SANHANES) revealed that in 2013 26% of the population faced hidden hunger while 28% already suffered from micronutrient deficiency diseases in South Africa [5]. The result from the survey proves that there is a need to address and mitigate the adverse effects that micronutrient deficiency diseases have on people.

Some scholars believe that the increase in micronutrient deficiency diseases is due to the drastic change in consumers’ food preferences [6–8]. Households nowadays prefer fast foods such as fried chips rather than healthy foods that could eradicate diseases and enhance the functioning of their bodies [7]. Such an attitude has exacerbated diseases such as obesity, diabetes, heart diseases, and stunted growth in children. In addressing this issue, scholars such as Mavengahama et al. [6] and Mbhenyane [8] have reported that African people need to go back to their roots and consume indigenous crops that once played a huge role in their diets in the past. World Health Organization (WHO) [9] has stated that these crops have a high concentration of nutrients required by the body to eradicate micronutrient deficiency diseases, enhance the quality of diets, and ultimately improve household food and nutrition security. However, their impact on people’s diets is minimal since these crops are less available in the market and undermined by people who perceive them as poor people’s food and unhygienic crops that grow in the wild.

Despite the negative critiques these plants have received from people over the years, indigenous leafy vegetables such as amaranth, nightshade, and spider plants can address micronutrient deficiency diseases because they have a high concentration of vitamins A, C, calcium, zinc, and iron required by the body for its normal functioning [10]. They also have characteristics that make them ideal for future research and investment to fight food insecurity, encourage climate-resilient agriculture, and develop a sustainable food system [11]. However, despite their perceived benefits, ILVs have been ignored by consumers who have replaced them with exotic crops such as cabbage and kale. Furthermore, few of these plants are economically utilized, due to factors such as human perceptions, lack of consumers’ awareness about their benefits, and cultural values [10].

Mbhenyane [8] further reported that there is a great potential for indigenous leafy vegetables to contribute to household food security if these leafy vegetables could be studied more extensively. However, their ability to contribute to household food security is hindered by the fact that they are not part of the food system; as a result, they are less available in the market. In addition, Mavengahama et al. [6] alluded that the inclusion of ILVs in the market as mainstream foods will increase their acceptance among urban and rural consumers. There are many studies that were conducted on the consumer acceptance of indigenous leafy vegetables, including studies by Van der Hoeven et al. [3], Sarah et al. [12], and Gido et al. [13]. On the other hand, other scholars such as Shayanowako, Morrissey [14], Mwadzingeni et al. [15] and Raheem et al. [16] have investigated the contribution of indigenous leafy vegetables to household food security. This implies that there is a knowledge gap of studies that links the two topics. Therefore, it is against this backdrop that the study seeks to assess the effect of consumers’ acceptance of indigenous leafy vegetables as well as their contribution to household food security.

## 2 Literature Review on the Acceptance of Indigenous Leafy Vegetables and Their Contribution to Household Food Security

Food security is a broad term defined in different ways by different scholars and organizations. However, the most cited definition of food security that of the 2006 World Food Summit which stated that “food security exists when all people, at all times, have physical and economic access to sufficient, safe and nutritious food that meets their dietary needs and food preferences for an active and healthy life” [17]. On the other hand, the Department of Agriculture, Forestry, and Fisheries (DAFF) [18] described food security as the ability of people to access adequate food. Their ability to access sufficient food is measured using four pillars: food availability, food access, food utilization, and food stability [17]. This implies that a household is considered food secure when all the pillars of food security are met. If not, the household is considered food insecure, increasing the chances of a household suffering from hunger and malnutrition. As a result, Mbhenyane [8] alluded that food-insecure households must increase their consumption of indigenous leafy vegetables since ILVs are easily accessible, nutritious, and widely available in South Africa. In addition to that, they have the potential to eradicate hunger and malnutrition-related diseases and increase dietary diversity and the dietary quality of a household.

Diversifying these leafy vegetables increases their chances of being a reliable, cheap food source, especially for food-insecure rural households. These leafy vegetables, such as amaranth, jute mellow, cassava, and cleome, grow relatively well in arable land where conventional crops fail to thrive. They can also provide at least two foodstuffs during their life cycle [19]. Akinola et al. [20] stated that ILVs have many desirable traits, such as that they are richer in protein when compared to exotic cash crops. According to World Health Organization (WHO) [9], a healthy diet protects the body against malnutrition, hidden hunger, and micronutrient deficiency diseases. However, an individual must consume at least 400 g of vegetables daily to achieve this. In this regard, indigenous leafy vegetables can provide most required nutrients since they comprise countless species of vegetables [20]. These nutrients include potassium, dietary fiber, folate, vitamin A, and vitamin C. Other benefits of producing these indigenous leafy vegetables are that they are easily dried up, processed, and canned for consumption in other seasons when they are less available [20]. This proves that ILVs can contribute to food security since they meet almost all pillars of food security as they are widely available and accessible, and promote food security, dietary diversity, and dietary quality among people, increasing their acceptance among rural and urban consumers.

Akinola et al. [20] further stated that the acceptance of these leafy vegetables is highly dependent on their physical and sensory characteristics, such as their appearance, odor, texture, and taste. These findings are similar to that of Maseko et al. [21] who stated that there was less acceptance of jute mellow because this leafy vegetable has a slippery texture. However, Shayanowako et al. [14] alluded that there is an increase in the acceptance of these leafy vegetables as people realize their various benefits. This implies that the acceptance of ILVs is dependent on their physical and sensory characteristics and on the nutritional and medicinal benefits of these leafy vegetables. Gido et al. [13] reported that the acceptance of ILVs among rural and urban consumers was equally high. The study further concluded that the high acceptance of ILVs among urban consumers could be attributed to increasing awareness of the nutritional benefits of consuming these leafy vegetables. However, Maseko et al. [21] alluded that these leafy vegetables are less accepted by rural young people who regard ILVs as unhygienic foods as they are obtained from the ground where there is soil and little stones which can be detected when eating them. They also resent a bitter taste found in them. This implies that in youth-headed households, these leafy vegetables have a minimal contribution to household food security due to their negative perceptions and attitudes toward ILVs. Matenge [12] reported that there was a high level of acceptance of ILVs in rural areas, which is because older people are knowledgeable about the preparation and cooking processes of these leafy vegetables.

Bvenura and Afolayan [22] stated that indigenous crops have minimal contribution to food security, especially in household food security of urban households. This is because the South African food database comprises 1472 food items, yet only 21 food items from 12 species of indigenous crops are part of the food system. This implies that ILVs are less available in the market, where they can be easily accessible by urban consumers who largely depend on the market to access food. Akinola et al. [20] stated that many ILVs like brassica are unfamiliar and, as a result, are categorized as underutilized leafy vegetables. This implies that their contribution to household food security is minimal. However, Mayekiso [23] stated that these leafy vegetables significantly contribute to the rural poor’s household food security. This is because ILVs are readily available and easily accessible in rural communities and are used mainly by rural consumers for medicinal and nutritional purposes. These findings align with that of Mavengahama et al. [6] who stated that these leafy vegetables could address household food security problems triggered by malnutrition and micronutrient deficiency diseases. Since they are easily available, accessible, and comprise a diverse set of leafy vegetables with high levels of nutrients.

Empirical studies have been conducted on the consumer acceptance of indigenous leafy vegetables [13,14,20,21] and nutritional value [9,20]. In the same vein, there are empirics on food security [6,8,21,23]. However, there is a dearth of studies on the nexus between consumer acceptance of ILVs and their contribution to food security. Therefore, this study aimed at providing new evidence-based information on how consumers’ choices of a certain type of ILV can affect household food security situation. The study will help to identify different determinants of consumers’ acceptance of ILV and food security. The results and recommendations arising from this study are expected to stimulate the consumption of ILVs for improved household food security outcomes.

In summary, the literature shows that indigenous leafy vegetables have the potential to contribute to household food security. However, some of these leafy vegetables are underutilized because they possess physical and sensory characteristics that consumers dislike. The literature further shows that rural people highly accept ILVs. This is because they are readily available and accessible in rural areas. However, less acceptance of ILVs by urban people is because they are less available in the market where they usually access food. These findings show that policymakers must promote the inclusion of ILVs in the market where they will be easily accessible to urban people who rely on the market to access food. They must develop policies that promote the inclusion of ILVs into the school curriculum and national food and nutrition security policy. Extension workers need to educate people about the importance of ILVs, nutritional benefits, and the ability to contribute to household food security. The literature available on indigenous leafy vegetables generalized the significance of these leafy vegetables in addressing household food security. This may have resulted in less inclusion of ILVs in strategies and policies aimed at improving household food security. Therefore, researchers need to provide extensive scientific evidence on the significance of these leafy vegetables and their ability to contribute to household food security.

## 3 Materials and Methods

### 3.1 The Description of the Study Area

The research was conducted in two of South Africa’s nine provinces, Limpopo and Mpumalanga. These provinces were chosen for the study because they have a high proportion of smallholder farmers who grow indigenous crops. These provinces comprise smallholder communal farmers who rely on agriculture and livestock farming for a living. Limpopo is located in northern South Africa and covers 125,754 km^2^, accounting for 10.2% of the country’s total area. This province has a population of 5.8 million people and is divided into five districts: Mopani, Vhembe, Capricorn, Waterberg, and Sekhukhune [24]. These are the districts where the study’s data was obtained because the people in these areas rely heavily on agriculture. This is also reflected in the fact that 89% of the citizens in this province work in agriculture. Mpumalanga is one of the provinces which relies heavily on agriculture, producing a wide range of fruits, vegetables, cereals, tea, and sugarcane. The production of these crops contributes significantly to Limpopo province’s economic growth and development [25]. It also includes 167 existing irrigation schemes that are used by small-scale farmers [26]. There are approximately 10,150 farmers in these small-scale irrigation schemes, with an average individual land holding of approximately 1.5 hectares per farmer.

Mpumalanga province was previously known as Eastern Transvaal. This province is in the northeastern part of South Africa. It is bounded to the north by Limpopo province and to the east by Swaziland. It accounts for approximately 6.5% of the country’s total land area. It has a population of 4.04 million people, with agriculture accounting for 72% of the workforce [24]. Mpumalanga receives 1000 mm of rainfall annually and has warm weather due to its elevation of 665 m above sea level. Amaranth, cowpea, African eggplant, okra, and pumpkin are indigenous crops grown there. Other foods grown by farmers in Mpumalanga include sugarcane, groundnuts, corn, potatoes, cotton, other vegetables, and a wide range of fruits, including mangoes and oranges in the subtropical Lowveld and peaches at higher elevations. Mpumalanga is known for its dairy cattle, beef, and wool production from sheep farming.

### 3.2 Data Types, Methods, Sources, Data Collection

The study used a quantitative method to analyze data collected on the key agricultural, food, and nutrition security indicators. The multistage random sampling method was employed to select people who would be part of the study [25]. This method allows individuals within a particular population to have an equal chance of being selected to participate in the study [25]. The multistage random sampling technique was employed because it is easy to use, cheap, and relatively easy to implement. In each site, the population was grouped according to similar characteristics they possess, such as socio-economic characteristics, outputs, sales, household size, and institutional factors. A total of 1520 respondents were selected from the two provinces of South Africa: Limpopo and Mpumalanga. The study used secondary data collected in 2016 by the South African Vulnerability Assessment Committee, led by the Secretariat hosted in the Department of Agriculture, Land Reform, and Rural Development. Although this data was collected in 2016, it is still relevant for determining the household food security of farmers and their acceptance of indigenous leafy vegetables. Data was computerized using Microsoft Excel, and the data set was described, summarized, and presented using descriptive statistics.

### 3.3 Conceptual Framework

**Figure 1:**
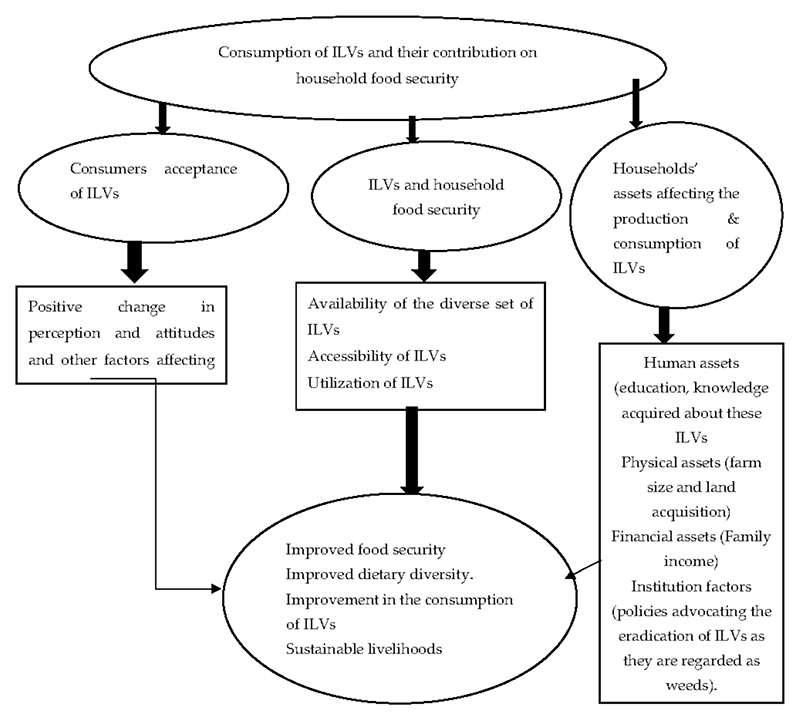
Conceptual framework for consumers’ acceptance of indigenous leafy vegetables and their contribution to household food security.

The concept employed in this study is developed on the consumption of ILVs and their impact on household food security. The framework explains how different factors are expected to affect consumers’ acceptance of ILV and their contribution to food security. The framework further explains the impact of ILVs on household food security if they are accepted, produced, and consumed by rural people. In a nutshell, the concept explains the benefits and outcomes of the potential contribution of ILVs on its consumers and smallholder farmers. The concept further outlines factors other than the consumption of ILVs that affect the production and consumption of these leafy vegetables. This includes the livelihood assets that may affect the production and consumption of ILVs. In the conceptual framework, the improvement of the livelihood assets results in sustainable livelihoods of smallholder farmers who produce these leafy vegetables. Figure 1 below shows a relationship between consumer acceptance, ILVs consumption, household food security, and its effect on the livelihoods of farmers.

### 3.4 Data Analysis

The study used an endogenous switching regression model to model the impact of indigenous leafy vegetables on consumers. The contribution of ILVs to household food security is determined by whether its consumers are food secure or food insecure. Following Lokshin and Sajaia [27], the equations for the two regimes are presented as follows:

(1) $$I^{*}=aZ+VI^{*}=a\text{Z}+V$$

(2) $$y_{1}=\beta_{1}X_{1}+u_{1}\;\text{if}\;I=1y_{1}=\beta_{1}X_{1}+u_{1}\;\text{if}\;I=1$$

(3) $$y_{0}=\beta_{0}X_{0}+u_{0}\;\text{if}\;I=0y_{0}=\beta_{0}X_{0}+u_{0}\;\text{if}\;I=0$$

where *y*_1_ and *y*_0_ represents food security and food insecurity of ILVs consumers, which take the value of 1 if household is food secure, and 0 if otherwise, *I* is the latent variable as defined in Equation (1), and *α, β*_1_ and *β*_0_ are vectors of the parameters to be estimated. *Z* is a vector of endogenous variables affecting consumers’ acceptance of indigenous leafy vegetables, *X*_0_ and *X*_1_ are vectors of exogenous variables. *v, u*_1_, *u*_0_ are error terms assumed to be jointly normally distributed with zero mean vector and the following covariance matrix.

(4) $$cov\left(v,u_{1},u_{0}\right)=\left[\begin{array}{ccc} \sigma_{u_{1}}^{2} & \sigma u_{1}u_{0} & \sigma_{u1}v\\ \sigma u_{1}u_{0} & \sigma_{u_{0}}^{2} & \sigma u_{0}v\\ \sigma u_{1}v & \sigma u_{0}v & \sigma_{v}^{2} \end{array}\right]$$

where $var\;(v)=\sigma^{2}v,var\left(u_{0}\right)=\sigma_{u_{0}}^{2},var\left(u_{1}\right)=\sigma_{u_{1}}^{2},cov\left(u_{1}u_{0}\right)=\sigma u_{1}u_{0},cov\left(u_{1},v\right)=\sigma u_{1}v$, and *cov*(*u*_0_*v*) = *σu*_0_*v*.

The variance $\sigma_{v}^{2}$ is assumed to be 1, as *α* can be estimated up to a scale factor. In addition, the covariance *σu*_1_*u*_0_ is equal to zero because *y*_1_ *y*_0_ are not observed together. The characteristics are related to selected bias; the structure of the error terms, *v*, of a selection Equation (1) is correlated with the error terms *u*_1_ and *u*_2_ of the generated Equations (3) and (4) with the expected values of *u*_1_ and *u*_2_ being conditional on the sample selection being non-zero.

The Household Food Insecurity Access Scale (HFIAS) is the other tool. This indicator is used to assess whether households have experienced problems with food access in the last 30 days [28]. This indicator consists of nine occurrence questions and nine frequency questions. These questions ask about the changes in household food patterns that may arise because of insufficient access to food [29]. As a result, HFIAS measures the level of food insecurity during the past 30 days as reported by the household. The results are then categorized into food secure, mild food secure, moderate food insecure, and severe food insecure [29]. The generic HFIAS questions help researchers determine a household’s degree of food insecurity over the past 30 days. The respondent determines whether this never happened, rarely (once or twice), sometimes (three to ten times), or often (more than ten times) in the past 30 days. Then, the HFIAS score variable is calculated for each household by adding the codes for each frequency of occurrence question. The maximum score for the household is 27 (if the household response to all nine occurrence questions was “often”) and the minimum score is 0 [28]. The higher the score, the higher the food insecurity experienced by the household. The lower the score, the lower the food insecurity faced by the household. Table 1 summarizes the variable names and definitions.

## 4 Results and Discussion

### 4.1 Descriptive Analysis of the Results

Table 2 shows that the mean age of ILV consumers was 47.33 while mean age for non-consumers of ILVs was 44.33. As for education, it was found to be statistically significant at 5%. The mean number of years spent by consumers at schools is 9.16, while the years spent by non-consumers of ILVs is 5.44. This simply means ILV consumers are more educated than non-consumers of these crops. The total output of crop production of these leafy vegetables was 2242.64 kg, while for non-consumers of ILVs was 717 kg. This means that the total output of ILVs was more than that of other crops. The possible explanation could be that they are readily available as they grow in the wild and uncultivated areas. Hence, their yield is high. Furthermore, the findings showed that 62% of these leafy vegetable consumers were females, while 38% were males. This is because females have a lot of knowledge about the preparation, cooking, and storage of indigenous leafy vegetables, which could explain this.

The descriptive statistics results from Table 3 show that 66 farmers produced and consumed ILVs. The results further revealed that 350 farmers did not produce and consume ILVs. However, those who did not produce ILVs but consumed them were 1059. The logical explanation for this is that farmers might rely on the market to access ILVs. This is in line with Senyolo et al. [30], who alluded that ILVs are sold in the informal market. As a result, they are easily accessible to consumers and farmers. These urban farmers focus more on producing staple crops than ILVs. As a result, they might purchase ILVs in the market for consumption purposes rather than producing them on their farms. However, 45 farmers produced ILVs and did not consume them. This is because the household member may decide to produce ILVs for commercial purposes instead of consumption. This is somehow in line with Mahlangu et al. [31], who stated that in some parts of Limpopo the ILVs are produced for commercial purposes. After that, they are sold in the market at a price ranging from R6.00 to R8.00.

### 4.2 Responses of Smallholder Farmers to Each Household Food Insecurity Access Scale Survey Question Option for the 2016/17 Season in Mpumalanga and Limpopo Province

The food security of rural households of Mpumalanga and Limpopo provinces was examined using an HFIAS score indicator as shown in Table 4. The results from this tool showed that the household response to occurrence questions was often “never”, particularly for the last two questions which focused on whether the household has gone a day or to bed without eating anything. This implies that the households in these two provinces are somewhat food secure or have experienced mild food insecurity. This is also evident in that a relatively small number of people responded “often” to the nine occurrence questions of HFIAS. The logical explanation could be that the household members experienced less food insecurity. Some respondents stated that they “rarely” experienced food insecurity. At the same time, some respondents mentioned that they “sometimes” encountered food insecurity in their households. In summary, these results show that both provinces, Mpumalanga and Limpopo, were food secure and experienced mild food insecurity. These results are contrary to that of Chinnakali et al. [32] who stated that their study found that 77.2% of the household were food insecure. However, they are somewhat in line with Oldewage-Theron and Egal [33], who found that the majority of their respondents were food secure (67%), while 4% of their respondents experienced mild food insecurity.

### 4.3 Occurrence of Food Insecurity by Household Characteristics Based on HFIAS Categories

**Figure 2:**
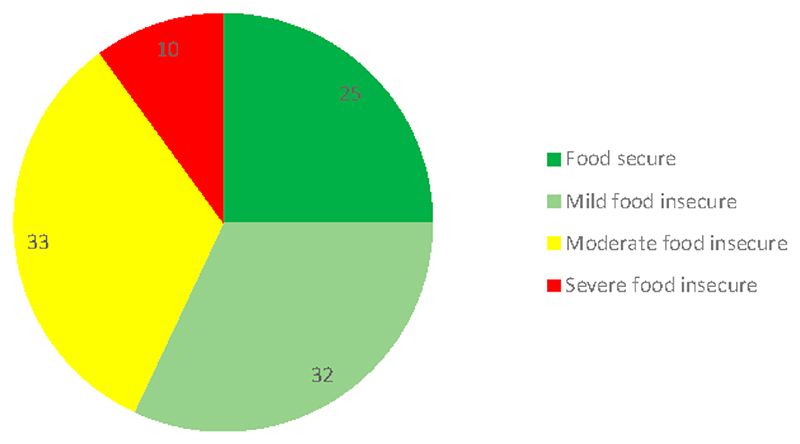
Count of households in Household Food Insecurity Access Scale (HFIAS) categories. Source: Authors’ analysis.

The results from HFIAS show that 25% of households were food secure while 32% were mildly food insecure (Figure 2). The results further reveal that 33% of households were moderately food insecure, while 10% of households experienced severe food insecurity. These results show that most households experience food insecurity in Limpopo and Mpumalanga provinces. This might imply that most of the households hardly consumed nutritious food such as indigenous leafy vegetables. This is in line with Mavengahama et al. [6], who alluded that the high consumption of ILVs leads to food and nutrition security.

### 4.4 Impact of Indigenous Vegetable Consumption on Household Food Security—Endogenous Switching Probit Model

The endogenous switching regression model results show that different variables influenced the consumption of ILVs. In this study, the consumption of ILVs or lack of consumption thereof determined whether the household is food secure or food insecure. The results from Table 5 below revealed that a few of the variables were statistically significant to the consumption of ILVs. This includes household size, wealth index, and ‘if a disabled member receives grants’. In this instance, the household size and wealth index variables positively influenced the consumption of ILVs, and both variables were statistically significant to the food security and food insecurity variables. The ‘if a disabled member receives grants’ variable was positive to the consumption of ILVs and statistically significant with the food security variable. However, it was not statistically significant with the food insecurity variable.

The household size variable positively influenced the consumption of ILVs and was statistically significant. This implies that as the household size increases, household members may grow various indigenous leafy vegetables for consumption and commercial purposes. These findings differ from those of Gido et al. [13] who stated that the consumption intensity of ILVs in large households is likely to decrease as they prefer staple crops over ILVs. These findings concur with Ayanwale et al. [34] who stated that a large household size indicates that greater quantities ILVs are required to feed everyone in the household. The results further show that consumption of ILVs influenced households’ food security and food insecurity. The logical explanation for the significant food-secure variable is that as the household size increases, the demand for food increases which then causes the household members to produce more indigenous leafy vegetables. In this instance, food insecurity may occur as the household size increases, so the household may be reluctant to diversify the ILVs they consume. The household quest can be on ensuring that everyone in the household is satiated without considering the importance of diversifying the ILVs they consume. This is somehow in line with Mavengahama et al. [6], who alluded that the diversification of these leafy vegetables is of paramount importance as it lowers the risk of diseases and food insecurity. This is contrary to Sekhampu [35], who stated that households of larger sizes are likely to suffer from food insecurity because they are often idle to diversify the foods they consume.

The gender of the household head negatively influenced the consumption of ILVs and was not statistically significant. However, it positively impacted household food security and was statistically significant. This implies that male-headed households may prefer fast foods as they do not require a tedious process to prepare and cook. Thus, resulting in food insecurity among the household members. These results are similar to Sanlier and Karakus [36], who alluded that male-headed households are likely to suffer from food insecurity as they rarely consume nutritious foods. These findings are contrary to that of Lee et al. [37] who indicated that, when compared to their male counterparts, females are more likely to suffer from food insecurity because they often sacrifice their quality of nutrition to protect their children from hunger.

The household head’s education negatively influenced the consumption of ILVs and was not statistically significant. However, it was positive and statistically significant on the food security variable. This implies that as the household member gets more educated, they tend to consume more nutritious food from various food groups such as vegetables, fruits, grains, and protein foods. This is in line with Akinola et al. [20], who stated that, as people become more educated, they often prioritize their well-being. Therefore, they consume healthy food from various groups to improve their food security status. However, Mungofa [38] found that as people get more educated their eating patterns also change. The study concluded that they consume unhealthy food perceived as luxurious food that matches their new status. Thus, resulting in food insecurity.

The marital status of the household head had a positive influence on the consumption of ILVs and was not statistically significant. Surprisingly, it was positive and statistically significant with the food secure variable. This implies that as the marital status of the household member changes, the household food security is positively influenced by those changes. These findings are in line with those of Sekhampu [35], who found that marital status was positive and statistically significant. The study concluded that as the male household head marries, the food patterns of the household change drastically. Thus, resulting in a positive change in the household food security status. However, Lee et al. [37] stated that the dissolution of marriage in couples (divorce) contributes considerably to food insecurity. This is because marriage dissolution leads to depression which ultimately leads to unhealthy eating patterns of fast foods.

Even though the household involvement in livestock production variable had a negative influence on the consumption of ILVs and was not significant, it had a positive impact on the food secure variable and was statistically significant. The logical explanation is that the household will likely consume nutritious foods from livestock production, such as milk, meat, beef, etc., to improve their food security status. This is in line with Jodlowski and Winter-Nelson [39], who stated that a household might expand livestock production to improve their food security through direct consumption of home-produced animal products. The study concluded that the expanded livestock activities also contribute indirectly to increased dietary diversity, enhancing the household’s food security status. However, Mayekiso et al. [40] stated that smallholder farmers might produce livestock for commercial purposes rather than for consumption purposes. This results in food insecurity over time since livestock production might be solely for commercial purposes and other animal services such as hauling and plowing, and not for the consumption purposes of the household.

The wage/salary variable had a negative impact on the consumption of ILVs and was not significant; however, it expectedly had a positive impact on food security and was statistically significant. This implies that the more the wage/salary increases, the more the household buys foods from various food groups in the market, such as vegetables and fruits and proteins. Thus, eradicating malnutrition and achieving food security within the members of the household. The South African government recommended R25.42 per hour to be a minimum wage/salary that can sustain households, therefore an increase in the minimum wage/salary result to improved household. These findings concur with Sekhampu [35], who stated that a salary increase results in food security as the household could afford to purchase more healthy food for their members. However, Lee et al. [37] alluded that some households may consume unhealthy foods as their salary increases.

The ‘if the household receives advice from government’ negatively affected the consumption of ILVs and was not significant. The government advice was found to have an unexpected positive impact on household food security and was statistically significant. The logical explanation is that consumers may receive advice from government extension services to improve the productivity of their cash crops, livestock farming, and non-farm activities. This is somehow in line with Adekunle [41], who stated that the government extension services contribute significantly to the food security of smallholder farmers who produce staple crops and ILVs since it provides them with information, resources, and knowledge on increasing the production of staple crops and ILVs. However, Shackleton [42] alluded that government advice may indirectly lead to food insecurity since it focuses on cash crops. Thus, promoting the eradication of other nutritious crops (especially ILVS) that grow in the field along with staple crops.

## 5 Conclusions and Policy Recommendations

Indigenous leafy vegetables play a significant role in household food security, especially in poor rural households; however, they are less consumed when compared to exotic crops. The study assessed the consumption of ILVs and their effect on household food security. The findings indicated that the consumption of indigenous leafy vegetables had a minimal impact on household food security. This is evident in that only a few variables of the consumption of ILVs were positive and significant (household size, wealth index, and if a disabled member receives a grant). Other variables were negative and had no statistical significance; however, most of the food security variables were positive and influenced the household food security status. Households relied on exotic cash crops and other nutritious foods besides ILVs to improve food security. There is a need to promote and raise awareness of the consumption and production of ILVs since these leafy vegetables are more nutritious than the staple crops that most households prefer. This is because ILVs are easily accessible and cost effective. More importantly, the consumption of ILVs has a potential to improve household food and nutrition security.

The findings from this study revealed that age, gender, and education negatively influenced the consumption of ILVs. These results highlight a need to promote awareness programs for ILVs where young people, males, and educated people (who are less fond of ILVs) will be informed about the importance and benefits of consuming ILVs. This can be achieved through awareness programs that pass knowledge regarding ILVs to consumers in languages they understand to promote acceptance of ILVs. Agricultural Extension Services must equally promote the consumption of exotic cash crops and ILVs. This can be done by disseminating information on the benefits of ILVs in health facilities such as hospitals and clinics. Also, circulating brochures with all the necessary information on these leafy vegetables could encourage people to consume more. Finally, policies can contribute by increasing the diversity of ILVs at retail outlets through diverse production.

## Figures and Tables

**Table 1:** Definitions and summary statistics of variables used in the endogenous switching probit Model.

Variable Name	Variable Definition
Age	Age of the household member (in years)
Gender	Gender of the household head (1 = male, 0 = female)
Marriage	Marital status of the household head (1 = married, 0 = otherwise)
Household size	Number of the family members (continuous)
Level of education	Level of education of the head in years (continuous)
HIV status	HIV status of the household head
Grants	If the household receive social grant
Wealth index	Principal component analysis was used to calculate wealth index
Livestock production	If participant owned any livestock (1 = yes, 0 = no)
Wage/salary	Salary/wage that participant received when working

**Table 2:** Socio-demographic characteristics of ILVs consumers.

Variables	Consumers’ Acceptance of ILVs	Mean	*F*-Value	Degrees of Freedom	*p*-Value
Age of the household head	Yes	47.33	1.009	129	0.314
	No	44.23		21.52	
Education of the household head	Yes	9.16	0.000	102	0.989
	No	5.44		17.14	
Total output of indigenous crops (KG)	Yes	2242.60	26.623	318	0.000 ***
	No	717.17		132.00	

*** Notes: statistically significant at the level of *p* < 0.01.

**Table 3:** Production and consumption of indigenous leafy vegetables.

		Consumption of Indigenous Leafy Vegetables		
		Yes	No	Total
Production of indigenous leafy	Yes	66	45	111
vegetables	No	1059	350	1409
Total		1125	395	1520

**Table 4:** Household Food Insecurity Access Scale survey among smallholder farmers in the 2016/2017 season in Mpumalanga and Limpopo province.

	Last 30 Days			
	Never	Rarely (1–2 Times)	Sometimes (3–10 Times)	Often (More than 10 Times)
Worry about not having enough food (%)	25	32	33	10
Do not eat your kinds of preferred food (%)	15	35	38	12
Limit the diversity/quality of meals (%)	20	40	18	22
Consume some foods that you really did not want to eat (%)	17	34	35	14
Limit eaten food portions (%)	26	34	31	9
Limit the number of meals (%)	30	32	28	10
No food to eat of any kind in your household (%)	57	22	17	4
Go to sleep at night hungry (%)	74	14	7	5
Go a whole day and night without eating anything (%)	81	10	6	3

**Table 5:** Impact of indigenous vegetable consumption on household food security—endogenous switching probit model.

	Consumption of ILVs			Food Secure			Food Insecure		
Variables	Coefficient	Standard Error	*p*-Value	Coefficient	Standard Error	*p*-Value	Coefficient	Standard Error	*p*-Value
Age of the household head	−0.003	0.003	0.366	0.001	0.003	0.772	−0.000	0.004	0.924
Household size	0.031	0.018	0.080 *	0.052	0.020	0.010 **	0.076	0.028	0.007 ***
Gender of the household head	−0.146	0.217	0.501	−0.481	0.419	0.252	0.909	0.471	0.054 *
If the household head resides in the farm	0.218	0.503	0.664	2.665	0.800	0.256	0.446	2.609	0.864
Education of household head	−0.389	0.591	0.511	2.188	1.183	0.064 *	1.704	5.804	0.769
Marital status of household head	0.059	0.775	0.939	4.672	2.073	0.024 **	1.770	4.874	0.717
Agricultural related training	−0.361	0.187	0.234	−1.098	0.334	0.235	1.184	0.632	0.254
Livestock production	−0.467	0.569	0.412	16.197	2.836	0.000 ***	1.609	2.890	0.578
Wage/salary	0.211	0.334	0.528	1.320	0.479	0.006 ***	1.052	3.335	0.752
Wealth index	0.361	0.189	0.256	−7.209	1.331	0.267	−1.372	0.801	0.666
Government advice	−0.189	0.202	0.348	0.887	0.360	0.014 **	0.558	0.455	0.220
Disability in the family	1.457	0.820	0.234	7.833	2.301	0.267	2.689	10.377	0.796
Member in a family with HIV	−0.225	0.429	0.601	−2.258	2.082	0.278	5.181	3.628	0.153
_cons	0.261	0.736	0.723	14.765	2.776	0.000 ***	−4.632	3.022	0.125
/athrho1	−2.769								
/athrho0	2.088								
rho1	−0.992								
rho0	0.970								
Prob > chi2	0.000								
LR (rho1 = rho0 = 0): chi2(2)	284.24								
Wald chi2(13)	815.51								
Log likelihood	−992.651								

Notes: ***, **, *—statistically significant at the level of *p* < 0.01, *p* < 0.05, *p* < 0.10, respectively. Wealth index— principal component analysis was used to calculate wealth index.

## Data Availability

Restrictions apply to the availability of these data. Data were obtained from the Department of Agriculture, Land Reform, and Rural Development (DALRRD) and are available from the South African Vulnerability Assessment Committee (SAVAC) secretariat with the permission of the Department of Agriculture, Land Reform, and Rural Development (DALRRD).
